# Hen Egg-White Lysozyme Crystallisation: Protein Stacking and Structure Stability Enhanced by a Tellurium(VI)-Centred Polyoxotungstate

**DOI:** 10.1002/cbic.201402597

**Published:** 2014-12-17

**Authors:** Aleksandar Bijelic, Christian Molitor, Stephan G Mauracher, Rami Al-Oweini, Ulrich Kortz, Annette Rompel

**Affiliations:** [a] Institut für Biophysikalische Chemie, Fakultät für Chemie, Universität Wien Althanstrasse 14, 1090 Wien (Austria) E-mail: annette.rompel@univie.ac.at; [b] School of Engineering and Science, Jacobs University P. O. Box 750 561, 28725 Bremen (Germany)

**Keywords:** Anderson–Evans, electrostatic interactions, hen egg-white lysozyme, polyoxometalates, protein structures

## Abstract

As synchrotron radiation becomes more intense, detectors become faster and structure-solving software becomes more elaborate, obtaining single crystals suitable for data collection is now the bottleneck in macromolecular crystallography. Hence, there is a need for novel and advanced crystallisation agents with the ability to crystallise proteins that are otherwise challenging. Here, an Anderson–Evans-type polyoxometalate (POM), specifically Na_6_[TeW_6_O_24_]**⋅**22 H_2_O (TEW), is employed as a crystallisation additive. Its effects on protein crystallisation are demonstrated with hen egg-white lysozyme (HEWL), which co-crystallises with TEW in the vicinity (or within) the liquid–liquid phase separation (LLPS) region. The X-ray structure (PDB ID: 4PHI) determination revealed that TEW molecules are part of the crystal lattice, thus demonstrating specific binding to HEWL with electrostatic interactions and hydrogen bonds. The negatively charged TEW polyoxotungstate binds to sites with a positive electrostatic potential located between two (or more) symmetry-related protein chains. Thus, TEW facilitates the formation of protein–protein interfaces of otherwise repulsive surfaces, and thereby the realisation of a stable crystal lattice. In addition to retaining the isomorphicity of the protein structure, the anomalous scattering of the POMs was used for macromolecular phasing. The results suggest that hexatungstotellurate(VI) has great potential as a crystallisation additive to promote both protein crystallisation and structure elucidation.

## Introduction

X-ray crystallography is currently the method of choice and the most widely used in the field of structural biology; most protein structures deposited in the protein data bank (PDB) were elucidated by this method (∼89 %). One of the biggest obstacles in structure determination is the phase problem, which is complicated in many cases because of the lack of homologous structures. The state of the art approach to address this problem is the introduction of anomalous scatterers by either soaking experiments or co-crystallisation. Initial phases can then be obtained by single or multiple isomorphous replacement (SIR, MIR) and single- or multi-wavelength anomalous dispersion (SAD, MAD).[1, 2] However, with the development of tuneable high-energy synchrotron radiation sources and sophisticated software, solving the phase problem has gradually become a minor problem. Thus, crystallisation now represents the bottleneck in the structure determination process.[3] Current methods in protein crystallisation are usually based on “trial and error”. Accordingly, the crystallisation of newly isolated and structurally unknown proteins can be very time consuming with no guarantee of success. Numerous chemical compounds and small molecules, referred to as additives, have been shown to have considerable effects on protein crystallisation. These additives are known to promote stabilisation and intermolecular, non-covalent crosslinks in proteins, thus facilitating lattice formation and hence crystallisation.[4] Bivalent cations are common additives; these facilitate correct folding for certain proteins and also act as inhibitors, thus stabilising enzyme conformation. Finding the appropriate additive for a particular protein is similarly a time-consuming trial-and-error process of. A “universal” additive for crystallisation (at least for a certain group of proteins) would therefore alleviate this bottleneck in obtaining suitable protein crystals.

Zhang et al. reported controlled protein crystallisation with the help of yttrium cations (Y^III^) to enable the crystallisation of bovine β-lactoglobulin (BLG) as high quality crystals belonging to a new space group.[5] The yttrium cations contributed to lattice formation by binding to surface-exposed acidic side chains. It was suggested that multivalent ions in combination with the rich phase behaviour of protein solutions at the boundaries of the liquid–liquid phase separation (LLPS) region would be a powerful approach for the crystallisation of acidic proteins, which have a low pI and therefore tend to be negatively charged at physiological pH.[5] LLPS occurs in supersaturated protein solutions thus forming drops within the solution and separated as a visible meniscus by adding a precipitant. These formed drops contain a higher protein concentration than the remaining solution because of the partition of precipitant/salt and protein into two coexisting phases (drop within solution).[6] Furthermore, it was shown that multivalent cations are able to induce “re-entrant condensation” (RC): with increasing cation concentration a solution of an acidic protein first becomes cloudy and then clear again by charge-inversion-induced redissolving of the protein. RC is a special salt-concentration-dependent phase behaviour in protein solutions: at low salt concentration long-range repulsions inhibit protein aggregation and thus retain the protein in solution, but with increasing salt concentration the negative surface charge is adsorbed by the positively charged cations thus leading to the reduction of the repulsion forces and enhancement of short-range attraction. These short-range attractions are dominant in the LLPS (LLPS region is reached during RC) thus leading to protein aggregation. The charge inversion during RC was demonstrated by electrophoretic light scattering, and the binding of cations to acidic side chains was modelled by Monte Carlo simulations. This rich phase behaviour was exploited to modulate the crystallisation of other acidic proteins, such as human serum albumin and ovalbumin.[7–9]

In this study we investigated polyoxometalates (POMs) as multivalent anions to induce the same effect for basic proteins as yttrium cations exhibit for acidic ones. We recently presented the first crystal structure of the latent and active forms of mushroom tyrosinase PPO4 (*Agaricus bisporus*).[10, 11] Tyrosinase crystals were only obtained in the presence of Na_6_[TeW_6_O_24_]**⋅**22 H_2_O as a crystallisation additive (PDB ID: 4OUA; ligand, (TEW)).

POMs are anionic metal oxide clusters of early transition metals in their high oxidation state. POMs are known for a variety of unique and applicable physical and chemical properties. They exhibit interesting features in terms of molecular composition, size, solubility, shape, charge density and redox potential.[12] Because of their unique structures and highly negative charge, POMs are capable of binding to positively charged regions of proteins by electrostatic interactions, and thus potentially rigidifying flexible loops or other regions in order to promote crystallisation.[14, 15] The most prominent example is the post-crystallisation treatment of the ribosomal 30S subunit with the tungsten cluster [P_2_W_18_O_62_]^6−^ to enhance the crystalline arrangement of the protein, and this led to improved resolution (from 9 to 3 Å) by stabilising regions of high protein flexibility.[15]

There are around 25 PDB entries that include POMs. In most of the associated reports, the POM and its interaction with the protein and/or its impact on crystallisation are described in scarce detail (or not at all). POMs are predominantly used as anomalous scatterers for SAD/MAD phasing. The major advantage of applying POMs rather than ordinary heavy scatterers is that a higher number of anomalous scatterers (bound together) results in enhanced anomalous signals, at even low resolutions.[16–20] This feature was very important for the structure determination of the bacterial large (50S) and small (30S) ribosomal subunits: tungsten clusters [PW_11_O_39_]^7−^, [PW_12_O_40_]^3−^ and [P_2_W_18_O_62_]^6−^ were used to elucidate the structures and to reveal the exit tunnel within the large subunit where polypeptides are believed to pass before leaving the complex.[21–23] However, the formation of some POM-protein complexes, which have been deposited in the PDB, was an experimental coincidence.[24–28] The oxoanions of some early transition metals (WO_4_^2−^, VO_4_^3−^, MoO_4_^2−^) are known to act as substrate analogues or inhibitors for a series of enzymes and have therefore been used as crystallization additives in order to investigate different enzyme conformations and/or inhibition mechanisms. The presence of these oxoanions under the given crystallization conditions initiated the self-assembly of the POMs, which then appeared as “by-products” in the crystal structure. Therefore, the binding of POMs to proteins and the resulting effects are rarely examined.

To our knowledge only eight POM-associated reports describing the positions and binding modes of the used POMs are published. Among them, self-assembly of two molybdenum-based POMs ([Mo_8_O_26_(Glu)N(His)H_*n*_]^(*n*−5)−^ and [Mo_6_O_27_H_*n*_]^(*n*−18)−^) and one tungsten-based POM ([W_3_O_10_H_*n*_N_3_]^(6−*n*)−^) in the molybdenum/tungsten storage protein showed precisely the POM coordination to the protein and the surrounding water molecules (PDB IDs: 4F6T (Mo clusters) and 2OGX (W cluster)).[29, 30] The PDB entries 4BVO, 4BVP, 3ZX0 and 3ZX2 describe in detail the binding modes between NTPDase1 of two different organisms (*Rattus norvegicus* and *Legionella pneumophila*) and a dodecatungstate ([W_12_O_40_H_2_]^6−^; PDB ID: 4BVO), decavanadate ([V_10_O_28_]^6−^; PDB ID: 3ZX2), octamolybdate ([Mo_8_O_28_]^8−^; PDB ID: 4BVP) and heptamolybdate ([Mo_7_O_24_]^6−^; PDB IDs: 4BVP, 3ZX0).[31,32] The remaining two entries, 1E59 and 1DKT, report the binding of vanadates [V_4_O_13_]^6−^ and [V_7_O_19_]^3−^ to cofactor-dependent phosphoglycerate mutase (dPGM) from *Escherichia coli* and human cyclin-dependent kinase subunit 1 (CksHs1), respectively. [V_7_O_19_]^3−^ was used as phosphate analogue (substrate) to investigate the CksHs1–ligand interaction, and [V_4_O_13_]^6−^ served as an inhibitor for elucidating the mode of inhibition of dPGM.[33, 34]

There has not been any report concerning the use of POMs to overcome the biggest hurdle in protein crystallography: crystallisation itself. For this reason, we investigated the use of the sodium salt of hexatungstotellurate(VI) Na_6_[TeW_6_O_24_]**⋅**22 H_2_O (TEW) as an additive in crystallisation experiments with hen egg-white lysozyme (HEWL) as a protein model for X-ray structure analysis.

HEWL, a small enzyme (14.3 kDa) is one of the most common model systems to study interactions with compounds (e.g., drugs, metals, etc.). The major advantages of HEWL are its ease of crystallisation across a wide range of conditions and its resistance to conformational changes.[35] HEWL has 17 positively charged (6 Lys, 11 Arg) and nine negatively charged residues (7 Asp, 2 Glu), thus leading to a net positive charge at pH below the isoelectric point (∼11). This positive charge makes HEWL even more attractive for investigation with the negatively charged TEW.

## Results and Discussion

### Overall structure of HEWL–TEW

Several HEWL–TEW crystals were harvested and investigated by X-ray diffraction experiments. The crystals diffracted to a resolution of 1.8 Å, and the structure was solved by a combination of molecular replacement (MR) and SAD, by exploiting the anomalous signals of the tungsten atoms. Data collection and refinement statistics are shown in Table 1.

**Table 1:** w=1>Data collection and refinement statistics

X-ray source	P11 (DESY)
**Crystal data**	
space group	*P*4_3_2_1_2
*a*, *b*, *c* [Å]	60.01, 60.02, 261.84
*α*, *β*, *γ* [°]	90, 90, 90
molecules per asymmetric unit	4
Matthews coefficient [Å^3^ Da^−1^]	2.03
solvent content [%]	39.5
max. resolution [Å]	1.81
**Data collection and processing**	
wavelength [Å]	1.02
resolution limits [Å]	29.25–1.81 (1.86–1.81)
no. of observed reflections	902 043 (80 356)
no. of unique reflections	46 104 (4520)
redundancy	19.6 (17.8)
*R*_p.i.m._^[a]^	0.044 (0.417)
*R*_merge_^[b]^	0.19 (1.70)
CC_1/2_	0.99 (0.59)
CC^*^	1 (0.86)
completeness [%]	99.99 (99.89)
<*I*/σ*I*>	15.72 (1.33)
**Anomalous signal**	
SigAno^[c]^	1.805
AnomCorr^[d]^ [%]	68
**Refinement statistics**	
resolution [Å]	29.25–1.81
reflections used	83 164
*R*_work_^[e]^ (%)	18.7
*R*_free_^[f]^ (%)	20.0
number of water molecules	646
average *B*-factor [Å^2^]	32.0
**Ramachandran plot^[g]^**	
most favoured regions [%]	96
additional allowed regions [%]	4
disallowed regions [%]	0
PDB ID	4PHI

[a] *R*_p.i.m._=Σ_*hkl*_{1/[*N*(*hkl*)−1]}^1/2^ Σ_*i*_|*I_i_*(*hkl*)−〈*I*(*hkl*)〉|/Σ_*hkl*_Σ_*i*_*I_i_* (*hkl*), where *I_i_*(*hkl*) is the *i*th observation of reflection *hkl*, and 〈*I*(*hkl*)〉 is the weighted average intensity for all observations of reflection *hkl*. [b] *R*_merge_=Σ_*hkl*_Σ_*i*_|*I_i_*(*hkl*)_*i*_−〈*I*(*hkl*)〉|/Σ_*hkl*_Σ_*i*_*I_i_*(*hkl*)_*i*_. [c] Mean anomalous difference in units of estimated standard deviation (|*F*(+)|−|*F*(−)|/*σ*); *F*(+) and *F*(−) are structure factors obtained from the merged intensity observations in each parity class. [d] Percentage correlation between random half-sets of anomalous intensity differences. [e] *R*_work_=Σ|*F*_calcd_|−|*F*_obs_|/Σ|*F*_obs_|×100, where *F*_calcd_ and *F*_obs_ are the calculated and observed structure factor amplitudes, respectively. [f] *R*_free_ is calculated for a randomly chosen 5 % of the reflections for each dataset. [g] Calculated by using COOT.

**Figure 1:**
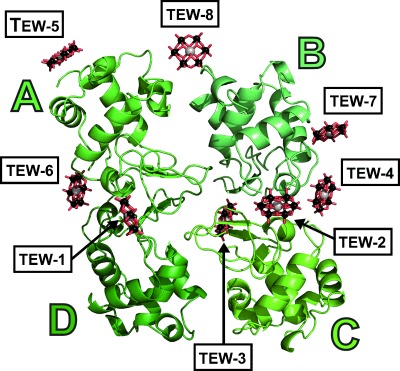
Asymmetric unit of HEWL–TEW structure. HEWL monomers are depicted as cartoons (monomer A–D in different shades of green); TEW molecules are shown as ball and stick (tellurium, grey; tungsten, black; oxygen, red). The catalytic sites of HEWL monomers point to the centre of the asymmetric unit.

**Figure 2:**
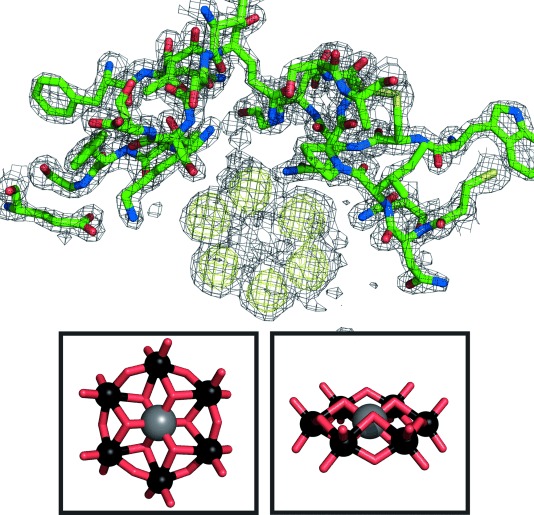
Anomalous difference map of HEWL–TEW structure. 2*F*_O_−*F*_C_ map (contoured at 1*σ*, grey) and anomalous map (contoured at 3*σ*, yellow) after model building and several refinement steps. Protein side chains are represented as ball and stick. Below: TEW molecule (ball and stick representation) from two different perspectives (tellurium, grey; tungsten, black; oxygen, red).

The HEWL–TEW complex crystallised in the *P*4_3_2_1_2 space group as a “tetramer” (chains A–D), and is only the second HEWL PDB structure with this protein stoichiometry (Figure 1). Eight TEW molecules (TEW-1–8) were identified per asymmetric unit. The exact positions of the tungsten atoms were determined by calculating an anomalous difference map (Figure 2), which revealed that the TEWs bind to positions in the tetramer in an arrangement that is not simply two POMs per monomer. The TEWs surround the tetramer and are mostly located at loop regions (no TEW within or in the vicinity of the asymmetric unit centre). Furthermore, no TEW was found in or near the catalytic cleft, including the critical residues Glu35 and Asp52, where, after substrate (peptidoglycan cell-wall) binding, Glu35 protonates the glycosidic oxygen thereby leading to cleavage of the glycosidic bond, while Asp52 stabilises the developing glycosyl–enzyme intermediate with a covalent bond (Asp52 is covalently bound to sugar rings).[36]

In order to investigate if the TEW molecules have any influence on HEWL conformation, the HEWL–TEW structure was superimposed on a series of HEWL PDB structures that differ in space group and bound ligands. Superimposition with monoclinic (PDB IDs: 2VB1, 1PS5), triclinic (1LKS), hexagonal (2FBB), orthorhombic (132L) and tetragonal (1IEE, 4B4E) HEWL structures revealed almost perfect structural matches, thus indicating that the protein in general is very stable towards conformational changes (RMSD_[residues]_=0.35–0.43 Å; RMSD_[Cα]_=0.25–0.40 Å); only two loops (Cys64–Leu75 and Asp101–Asn103) showed slightly variable conformations. Thus, POM did not alter the physiological conformation of the protein, thus further validating its use in protein crystallisation. Moreover, the catalytic centre was not affected, so no inhibition of the enzyme by TEW is expected. The resolution of the HEWL–TEW crystals was compared to HEWL structures in the PDB to determine if TEW enhanced crystal quality. The HEWL–TEW resolution (1.8 Å) is approximately the average for HEWL, as most of the deposited structures exhibit resolutions of 1.0–2.0 Å. Therefore, the use of POMs did not enhance crystal quality in terms of resolution.

### Crystal packing

**Figure 3:**
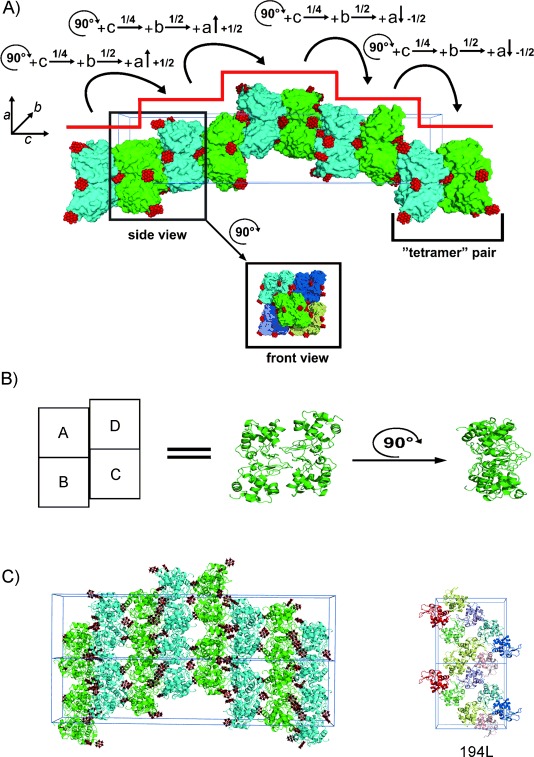
Crystal packing of the HEWL–TEW structure. A) Crystal packing in a 1×1×1 supercell. The unit cell lattice is shown in blue. HEWL “tetramers” are depicted in surface representation to show the parallel “tetramer” pairs. Different colours (green and cyan) distinguish adjacent “tetramers”. TEWs are illustrated as red spheres. Inset: “tetramer” stacked on four other “tetramers” with its opposite side (i.e., not involved in pair formation); three of the four “tetramers” are from the adjacent unit cell (different colours) and therefore are not visible in the supercell illustration. Curved arrows represent the operations to convert one “tetramer” pair into another pair. Red lines indicate the “rostrum”-like arrangement in the unit cell. B) Asymmetric unit consisting of a tetramer (chain A–D). Rotation by 90° of the tetramer is depicted to show the side view of the asymmetric unit (used in the picture below). C) Crystal packing of HEWL–TEW in a 1×2×1 supercell and the tetragonal reference structure of HEWL (PDB ID: 194L, right).[38] Unit cell lattices are shown in blue, protein molecules from HEWL–TEW and the reference structure are represented as cartoons; TEW molecules are shown as ball and stick (tellurium, grey; tungsten, black; oxygen, red). HEWL–TEW colours (green and cyan) indicate the stacking to neighbouring protein layers mediated by TEW; colours (red, yellow, green, magenta, blue, pink and cyan) of the reference structure distinguish different asymmetric units consisting of one monomer.

The HEWL–TEW complex crystallised in the tetragonal *P*4_3_2_1_2 space group, which is the most abundant space group for HEWL in the PDB (193 of 249 entries). However, the cell constants differ significantly from PDB structures of the same space group. The HEWL–TEW crystal shows an unusually long *c*-axis (261.84 Å; average for tetragonal HEWL, ∼40 Å), thus indicating a significant extension along the *c*-axis. In addition, the tetrameric arrangement of the HEWL monomers in the asymmetric unit is unique for this protein. PISA analysis (proteins, interfaces, structures and assemblies)[37] revealed that there are no specific interactions that could lead to a stable quaternary assembly of HEWL in solution (hence “tetramer” is enclosed in quotation marks in this text; this is not a biological tetramer). The “tetramer” is stabilised by crosslinks between individual monomers to maintain this unique arrangement. Crystal packing analysis revealed that the unit cell can be described as an assembly of four “tetramer” pairs, with the first and the fourth pairs overlapping with adjacent unit cells (Figure 3 A). “Tetramers” forming a pair are parallel to each other but differently orientated (displaced relative to each other by 90°) sharing one interface. This interface is the result of two monomers being crosslinked by four TEWs, with each monomer providing two POM clusters (TEW-1 and -2). Thus, one side of each “tetramer” is involved in the formation of the respective “tetramer” pair, whereas the other side is linked to four other “tetramers”, whose opposite sides are also involved in “tetramer” pair formation, and so on. The interaction of the opposite side with the four adjacent “tetramers” (see inset of Figure 3 A) is also mediated by TEW molecules. The rotation of one “tetramer” (90°) and subsequent translation (by 1/4*c* along the *c*-axis, ±1/2*a* along the *a*-axis and ±1/2*b* along the *b*-axis) produces the next “tetramer” pair in the packing (the ± sign reflects the fact that when starting the packing from the origin of the unit cell two “tetramer” pairs can be positioned in +*a*-axis direction reaching the edge of the unit cell; the next pairs have then to be positioned in a −*a* direction to fulfil correct packing, thereby resulting in an arrangement that resembles a winner's rostrum). The TEWs are distributed in such a way that the formation of stable “tetramer” pairs in the *c*-axis is preferred, and thus leads to the reported growth in this direction. Several of these “tetramer” pairs are connected and form protein layers that stack with TEWs like bricks with mortar. Figure 3 C compares the crystal packing of HEWL–TEW with that of the tetragonal HEWL structure used as the model for molecular replacement (PDB ID: 194L; RMSD_[Cα]_=0.285).[38] The difference in the packing is clearly visible and seems not to be related, probably because of their distinct protein stoichiometries (tetramer vs. monomer). The differences in cell constants and crystal packing led to the conclusion that TEW had induced a new crystal form.

### POM binding mode

**Figure 4:**
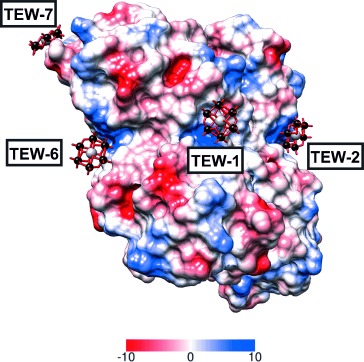
Electrostatic (Coulomb) potential surface presentation of HEWL–TEW. TEW molecules are illustrated as ball and stick (tellurium, grey; tungsten, black; oxygen, red). The figure shows clearly the binding of TEW molecules to protein pockets exhibiting a positive potential (blue). An additional protein surface would also bind to POM with a positively charged pocket from the solvent exposed site (not illustrated).

Eight TEW molecules per HEWL “tetramer” are bound at positively charged pockets formed by at least two protein monomers, as shown by the coulombic surface of the protein “tetramer” (Figure 4). The predominant interactions between [TeW_6_O_24_]^6−^ anions and the protein are electrostatic interactions and hydrogen bonds. The latter are formed because crystallisation occurred at pH 4.8, so all basic side chains involved in the binding are protonated (pI (Lys)=9.8, pI (Arg)=11.5).

**Figure 5:**
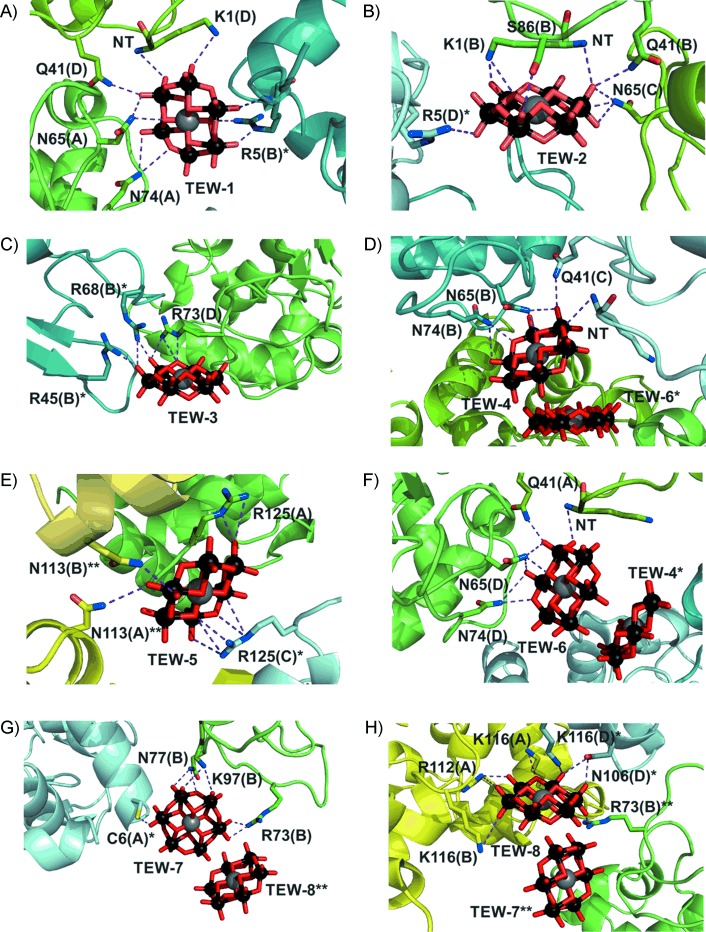
TEW binding sites. A–H) Binding modes of all TEW molecules (TEW-1 to -8). Involved protein side chains are illustrated as sticks (nitrogen, blue; oxygen, red; sulfur, yellow); the rest of the protein structure is depicted as a cartoon (20 % transparency). Bonds are shown as purple dashed lines. Colours (green, cyan and yellow) of the protein backbones differentiate monomers from different “tetramers”; different shades of a colour represent distinct chains within the same “tetramer”; parentheses after residue numbers also indicate chain; */**/*** denote distinct “tetramers”. NT: N terminus.

Figure 5 shows the interactions for all TEWs in detail (further descriptions in Table S1 and Figure S1 in the Supporting Information). For clarity, not all possible interactions are depicted, and water molecules are omitted (water molecules within the structure did however form hydrogen bonds with terminal oxygens of the polyoxotungstate). In general, all TEW molecules are bound to positively charged basic (Arg and Lys) or polar/hydrophilic residues (Asn and Gln), with distances from 2.0 to 4.1 Å (strong hydrogen bonds to weak electrostatic interactions).[39] Importantly, the TEWs interact mainly with side chains in flexible regions, including β-bridges (single-pair β-strand hydrogen bond formation), bends (regions with high curvature) and turns between secondary structural elements.[40] Six of the residues that interact with TEWs are in α-helices, with only one in a β-strand (Table S1).

The POMs are bound differently (Figures 1 and 5): TEW-5 and -8 have the most binding partners and interact with four HEWL chains. TEW-1 and -2 crosslink three HEWL monomers and in addition stabilise the very flexible N terminus of two monomers. TEW-3, -4 and -7 interact with two monomers; the only TEW not crosslinking different HEWL monomers is TEW-6 (note that TEW-6 rigidifies one N terminus).

Besides their different binding modes, the TEWs also have very different occupancies and associated mean *B*-factors (Table S2), thus suggesting multiple positions/orientations or conformations. TEW-1 has the highest occupancy (1.00) and a correspondingly low temperature factor (35.5 Å^2^; mean *B*-factor 32 Å^2^), thus suggesting fully occupied and highly ordered TEW-1 localisation. TEW-3, -4, -6 and -8 have lower occupancies, however, with clearly defined electron densities. TEW-4 and -6 are very close (∼2.5 Å); this suggests that both POMs are not present simultaneously (Figure 5 D and F) because of electrostatic repulsion. Thus, the low occupancies arise from the fact that both POMs are randomly positioned throughout the entire crystal. The same applies for TEW-7 and -8 (2.6 Å between them; Figure 5 G and H).

TEW-7 exhibits a very high temperature factor (∼204 Å^2^), which does not necessarily correspond to pronounced motion of the POM but to alternative conformations and/or orientations. Depending on its orientation, TEW-7 is bound more strongly to one of the two symmetry-related monomers. Refinements of the two different positions of TEW-7 (and TEW-5)—first bound to one monomer and then to the symmetry-related one—led to slightly different results, and indicated that both positions are possible for TEW-7. Accordingly, TEW-7 “moves” back and forth between the adjacent “tetramers” with different orientations. The results show that the TEW anions interact with positively charged pockets or surfaces, thus enabling new crystal packing contacts by connecting otherwise mutually repulsive surfaces. This promotes stable crystal lattice formation. The inhomogeneous distribution of basic and polar/hydrophilic residues on the HEWL surface provides regions that are favoured by negatively charged molecules, such as the TEW polyoxometalate. This has also been demonstrated for HEWL with different monovalent anions (Cl^−^, Br^−^, NO_3_^−^, SCN^−^, etc.) thus suggesting 14 specific anionic sites.[41] TEW shares three anionic sites that were previously identified: site 3 (TEW-4 bound to Asn113, Figure 5 E), site 4 (TEW-1, -4 and -6 bound to Asn66 and Asn74, Figure 5 A, D and F) and site 14 (TEW-3 bound to Arg45 and Arg68, Figure 5 C). All these sites are believed to be predominantly observed for the *P*4_3_2_1_2 space group, thus suggesting that anion binding to these sites promotes this tetragonal space group.

TEW–solvent interactions do not play an important role in the HEWL–TEW structure, as TEW interacts with the protein side chains (on the basis of interaction distance). However, in other structures like the molybdenum storage protein loaded with octamolybdate clusters, the interactions between the clusters and the protein is partially mediated by the solvent, with multiple hydrogen bonds stabilising the protein and POM.[29] Thus, POMs are able to interact directly with the protein or indirectly via solvent molecules.

A recently published structure demonstrates a covalent interaction of an octamolybdate with a serine side chain of LpNTPDase1 and a histidine of its His_6_-tag.[31] The latter is of particular interest because the octamolybdate rigidifies the typically very flexible His-tag at the N terminus. This certainly had a strong positive influence on the crystallisation. In this study we observed a similar situation: TEW-1, -2, -4 and -6 stabilisation of the mobile N termini of single monomers facilitated crystallisation. No covalent bond was observed between TEW and HEWL.

Another factor that plays an important role is size/shape of the POM. By binding two repulsive surfaces, the POM acts as a spacer between two monomers, thus overcoming steric hindrance or clashes between other scaffolds of the monomers. This space might reduce the long-range repulsion between protein monomers and simultaneously increase the probability of short-range attraction, thus leading to the initiation of crystal nucleation. In addition, the considerably higher negative charge is delocalised over a larger area (compared to other anions), thus allowing the TEW anion to neutralise more basic/hydrophilic protein residues. The interplay of this high charge and an appropriate size of the POM could facilitate protein crystallisation.

### Crystallisation with TEW at the LLPS region

Yttrium cations have induced crystallisation of high quality crystals within the LLPS.[7–9] Muschol and Rosenberger determined the phase diagram and the associated LLPS region (including phase boundaries) for supersaturated solutions of HEWL with NaCl, and showed that within (or close to) the LLPS, nucleation and crystallisation rate are enhanced.[42] By using this phase diagram, the LLPS of HEWL was reached, and its rich phase behaviour was exploited for crystallisation with TEW. A condition was found where nearly exclusively single crystals of HEWL grew in the presence of TEW (130 mg mL^−1^ protein, 7 % NaCl (*w/v*), 20 mm TEW, 0.1 m sodium acetate (NaOAc), pH 4.8). This was reproducible, and always resulted in single crystals suitable for X-ray diffraction. The control approach (same conditions but without TEW) led to clear drops or gelation of the protein.

**Figure 6:**
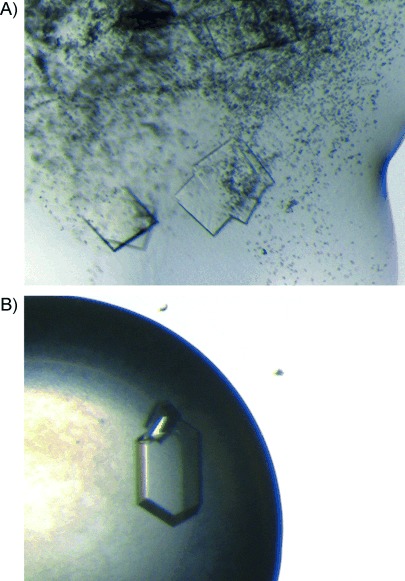
HEWL–TEW crystals by two approaches. Both crystals were obtained under same conditions (130 mg mL^−1^ HEWL, 0.1 m NaOAc pH 4.8, 5–9 % NaCl (*w/v*) and 20 mm TEW). A) Crystals grow although precipitation occurred; the amount of precipitation decreased with time. B) A visible LLPS formed (yellowish ring). The crystal is at the phase boundary.

During the preparation of the crystallisation drops with TEW, a precipitate quickly formed, but this was progressively re-dissolved by developing a visible LLPS (drop within solution), slight precipitation or gelation (Figure 6). In all cases, crystals were formed, even in drops where gelation or slight precipitation occurred (Figure 6 A). It had been assumed that nucleation is arrested in gelatinous protein solutions. This observation was also made for acidic proteins with yttrium cations: charge-inversion-based re-entrant condensation was probably induced by compensation of the protein's negative charge with cations.[8] Therefore, the zeta potential of HEWL (4 mg mL^−1^, 280 μm) in NaOAc (5 mm, pH 4.8) was measured with varying TEW concentration (0–1.5 mm). The potential of the HEWL–TEW solutions reversed (from positive to negative) with increasing TEW concentration (Figure S2). The charge inversion took place at a TEW concentration of 0.35–0.45 mm (1.3 to 1.6 excess of TEW). In the crystallisation experiments, the TEW concentration was about twice that of the protein (20 mm TEW, 9.5 mm HEWL) thus leading immediately to turbidity of the solution. The values presented here are rough estimates (hydration and dispersion forces were ignored as there was no available model), but are sufficiently accurate to demonstrate the inversion of electrophoretic mobility with increasing TEW concentration.

Zhang et al. showed multivalent-cation-induced re-entrant condensation (including charge inversion close/within the LLPS region) for several acidic proteins but not for HEWL.[43] This, however, relied on the fact that HEWL is a basic protein (pI ∼11) and thus the used cations were not sufficiently attracted by the predominantly positively charged surface. HEWL was shown to exhibit charge inversion with increasing concentrations of monovalent anions from the Hofmeister series (Cl^−^, NO_3_^−^, Br^−^, I^−^, ClO_4_^−^, SCN^−^) at pH 9.4 (i.e., less than the pI of HEWL).[44] Moreover, a very recent study showed that the dynamics of HEWL solutions demonstrate not only the occurrence of charge inversion but even re-entrant condensation.[45]

Here we explored all TEW–protein interactions induced at the LLPS region under the employed conditions. Notably, the phase behaviour of protein solutions within (or close to) the LLPS boundary is very complex, and small changes in the experimental conditions (e.g., pH, temperature or protein and salt concentration) can affect the location in the protein phase diagram; therefore a transition at these regions is a complex interplay and leads to different results.[46]

## Conclusion

We applied Na_6_[TeW_6_O_24_]**⋅**22 H_2_O as a crystallisation tool. During crystallisation, TEW induced charge inversion of the protein surface within the LLPS region, thereby leading to a reduction of repulsive forces between HEWL molecules and thus to the formation of new crystal contacts. TEW was demonstrated to have positive effects on HEWL crystallisation by enhancing protein–protein interactions during crystallisation by the stacking of protein monomers and layers. There are three advantages to TEW in comparison to other mono- or multivalent ions: 1) it has a higher negative charge distributed over a large size, thus, TEW is more strongly adsorbed onto positively charged protein patches than less-charged and polarisable mono- and multivalent anions; 2) its large size provides a certain distance between crosslinked monomers thus reducing the probability of clashes between other parts of the monomers, whereas crosslinking by small anions results in a shorter distance between interacting monomers and enhanced clashing; and 3) the anomalous signals originating from its tungsten atoms can be used to solve the phase problem, even at low resolution, by acting as “superatoms”.[16] In addition, unlike other POMs mentioned in this report, TEW is stable over pH 4–8. Protein crystallisation was achieved over pH 2–10 (most proteins crystallised at pH 4–9), thus TEW covers a broad protein crystallisation pH range. TEW therefore represents a powerful tool for the crystallisation of particularly basic proteins and could be the key to accessing new structures of proteins that are otherwise not crystallisable.

## Experimental Section

**Materials:** Hen egg-white lysozyme was purchased as lyophilised powder from Sigma–Aldrich; Na_6_[TeW_6_O_24_]**⋅**22 H_2_O was synthesised as described elsewhere.[11] All other chemicals were purchased from Carl Roth.

**Sample preparation:** In order to initiate liquid–liquid phase separation the sample was prepared as reported by Muschol and Rosenberger.[42] Briefly, lyophilised HEWL was reconstituted in NaOAc (0.1 m, pH 4.8) and NaCl (5–9 %, *w/v*). The pH was adjusted to 4.8 after protein reconstitution. The protein solution was applied to a Superdex 200 10/300 GL column (GE Healthcare) equilibrated with the above buffer. Protein monomer fractions were pooled and concentrated (maximum, 135 mg mL^−1^) in a Vivaspin 20 (cut-off 10 kDa; Sartorius, Göttingen, Germany).

**Protein crystallisation:** Crystals were obtained by the hanging drop vapour diffusion method at 293 K in a 15-well EasyXtal plate (Qiagen). HEWL protein solution (1 μL, 130–135 mg mL^−1^) was mixed with reservoir solution (1 μL) and Na_6_[TeW_6_O_24_]**⋅**22 H_2_O (1 μL, 20 mm), equilibrated against reservoir solution (500 μL; NaOAc (0.1 m, pH 4.8) NaCl (5–9 %, *w*/*v*)). Crystals appeared between 5 and 30 days. The best crystals grew at 7 % NaCl and in the presence of TEW.

**Data collection and processing:** Single crystals were flash cooled in liquid nitrogen (cryo-protectant solution contained NaOAc (0.1 m, pH 4.8), NaCl (11.3 %, *w*/*v*) and glycerol (15 %)). X-ray diffraction data was collected on Beamline P11 (*λ*=1.02 Å) at DESY (Hamburg, Germany) using a PILATUS 6M detector. X-ray diffraction data were collected at 1.81 Å resolution (oscillation range 0.1°, exposure time 0.1 s, crystal to detector distance 290 mm). The obtained data were processed with XDS,[47] and the structure was solved with programs from the CCP4[48] and PHENIX[49] suites. Data collection and refinement statistics are summarised in Table 1.

**Structure analysis and refinement:** The structure was solved by molecular replacement and single-wavelength anomalous dispersion (MR-SAD). Anomalous signals were provided by the tungsten atoms of TEW (*f* *′*=−7.74, *f* *′′*=12.23). The crystals belonged to the *P*4_3_2_1_2 space group with unit-cell parameters of *a*=60.01 Å, *b*=60.02 Å and *c*=261.84 Å (*α*=*β*=*γ*=90°) as indicated by POINTLESS (CCP4 suite). The asymmetric unit contained four protein molecules, and the solvent content was 39.5 % with a Matthew's coefficient of 2.03 Å^3^ Da^−1^ (according to xtriage (PHENIX suite) by calculating the solvent content from the protein sequence excluding TEW molecules). Initial phases were obtained by PHASER[50] with PDB ID: 194L[38] (without ligands) as the search model. Refinement of the resulting MR model was limited to *R*_free_ values of about 35–40 %, thus leading to blurred electron density maps, although the POMs in the maps were clearly visible. Thus, MR-SAD phasing was performed by applying AutoSol Wizard.[51] Phase calculations were performed by SOLVE, and density modification was performed by RESOLVE (part of AutoSol, PHENIX). Afterwards automated model building and refinement was carried out by AutoBuild (PHENIX). The resulting model was manually improved in Coot[52] and refined with phenix.refine (PHENIX). During the final refinement steps TEW molecules were inserted into the electron density map by using a restraint file generated by phenix.reel and phenix.elbow from a previous crystal structure of TEW.[53] After refinement including the TEWs, large negative and positive densities appeared at some TEWs (especially at terminal oxygen atoms) with high *B*-factor values. Therefore, the occupancies and *B*-factors of the TEWs were separately refined (by applying many refinement steps) starting with different values for occupancy and *B*-factor, until convergence was reached. Refined structures with similar low *R* values were selected, and the refined occupancies and *B*-factors were analysed. Average values were then taken for the occupancies and *B*-factors, (Table S2). The final X-ray structure was deposited to the PDB (ID: 4PHI).

## Supporting Information

As a service to our authors and readers, this journal provides supporting information supplied by the authors. Such materials are peer reviewed and may be re-organized for online delivery, but are not copy-edited or typeset. Technical support issues arising from supporting information (other than missing files) should be addressed to the authors.

miscellaneous_information
