# Supplementary material for: Hen Egg-White Lysozyme Crystallisation: Protein Stacking and Structure Stability Enhanced by a Tellurium(VI)-Centred Polyoxotungstate
Source: Chembiochem. 2014 Dec 17;16(2):233–41. doi: 10.1002/cbic.201402597 (PMC4498469; doi:10.1002/cbic.201402597)
Supplement: Supplementary file 1 — miscellaneous_information [file cbic0016-0233-sd1.pdf]

CHEMBIOCHEM

Supporting Information

© Copyright Wiley-VCH Verlag GmbH & Co. KGaA, 69451 Weinheim, 2015

Hen Egg-White Lysozyme Crystallisation: Protein Stacking and Structure Stability Enhanced by a Tellurium(VI)-Centred Polyoxotungstate

Aleksandar Bijelic,[a] Christian Molitor,[a] Stephan G. Mauracher,[a] Rami Al-Oweini,[b, c] Ulrich Kortz,[b] and Annette Rompel*[a]

cbic_201402597_sm_miscellaneous_information.pdf

Supporting Information

TEW-protein interactions

In Table S2 all interactions are summarized between the TEW molecules and HEWL. All TEW oxygens are numbered and shown in Figure S1.

Table S1. TEW-protein interactions.

TEW no.	Chain ID	AU[a]	Coordinating TEW oxygen	Coordinating protein residue	Distance (Å)	TEW no.	Chain ID	AU[a]	Coordinating TEW oxygen	Coordinating protein residue	Distance (Å)
1	A	*	O 13	Asn 65 (Nδ)	3.0	5	A	*	O 5	Arg 125 (Nε)	2.4
	A	*	O 16	Asn 65 (Nδ)	2.9		A	*	O 5	Arg 125 (Nη)	3.8
	A	*	O 20	Asn 74 (Nδ)	3.1		C	**	O 1	Arg 125 (Nε)	3.8
	A	*	O 24	Asn 74 (Nδ)	3.6		C	**	O 8	Arg 125 (Nε)	4.0
	D	*	O 15	Lys 1 (Nα)[b]	3.6		C	**	O 10	Arg 125 (Nε)	3.6
	D	*	O 16	Lys 1 (Nα)[b]	3.8		C	**	O 12	Arg 125 (Nη)	3.6
	D	*	O 5	Lys 1 (Nζ)	3.4		C	**	O 12	Arg 125 (Nη)	3.9
	D	*	O 16	Gln 41 (Nε)	2.9		C	**	O 18	Arg 125 (Nη)	3.2
	B	**	O 17	Arg 5 (Nη)	3.7		A	***	O 23	Asn 113 (Nδ)	3.6
	B	**	O 19	Arg 5 (Nη)	3.3		A	***	O 23	Asn 113 (Nδ)	3.8
							B	***	O 24	Asn 113 (Nδ)	3.9
2	B	*	O 21	Lys 1 (Nα)[b]	3.3						
	B	*	O 1	Lys 1 (Nζ)	3.4	6	A	*	O 14	Lys 1 (Nα)[b]	3.9
	B	*	O 8	Lys 1 (Nζ)	2.3		A	*	O 15	Lys 1 (Nα)[b]	3.8
	B	*	O 10	Lys 1 (Nζ)	3.8		A	*	O 16	Lys 1 (Nα)[b]	3.6
	B	*	O 6	Ser 86 (Oγ)	2.9		A	*	O 15	Gln 41 (Nε)	2.6
	B	*	O 24	Ser 86 (Oγ)	3.8		A	*	O 2	Asn 65 (Nδ)	2.8
	B	*	O 21	Gln 41 (Nε)	3.2		A	*	O 5	Asn 65 (Nδ)	3.7
	C	*	O 17	Asn 65 (Nδ)	3.7		A	*	O 15	Asn 65 (Nδ)	3.1
	C	*	O 21	Asn 65 (Nδ)	3.0		A	*	O 3	Asn 74 (Nδ)	3.1
	C	*	O 22	Asn 65 (Nδ)	3.3		A	*	O 5	Asn 74 (Nδ)	3.7
	D	**	O 5	Arg 5 (Nη)	3.4		A	*	O 9	Asn 74 (Nδ)	4.0
3	D	*	O 7	Arg 73 (Nε)	4.0	7	B	*	O 20	Arg 73 (Nη)	4.0
	D	*	O 11	Arg 73 (Nε)	4.0		B	*	O 23	Arg 73 (Nη)	2.0
	D	*	O 17	Arg 73 (Nε)	2.8		B	*	O 24	Arg 73 (Nη)	2.5
	D	*	O 17	Arg 73 (Nη)	3.2		B	*	O 24	Arg 73 (Nε)	3.7
	D	*	O 22	Arg 73 (Nη)	3.1		B	*	O 4	Asn 77 (Nδ)	3.2
	B	**	O 21	Thr 47 (Oγ)	3.7		B	*	O 6	Asn 77 (Nδ)	3.5
	B	**	O 23	Arg 45 (Nη)	2.4		B	*	O 5	Lys 97 (Nζ)	4.1
	B	**	O 22	Arg 68 (Nη)	2.7		B	*	O 15	Lys 97 (Nζ)	3.5
	B	**	O 23	Arg 68 (Nη)	4.1		A	**	O 9	Cys 6 (Sγ)	3.7
4	B	*	O 1	Asn 65 (Nδ)	3.1	8	A	*	O 6	Arg 112 (Nη)	4.0
	B	*	O 8	Asn 65 (Nδ)	2.9		A	*	O 8	Arg 112 (Nη)	2.8
	B	*	O 4	Asn 74 (Nδ	3.0		A	*	O 10	Lys 116 (Nζ)	3.8
	B	*	O 6	Asn 74 (Nδ	3.6		B	*	O 5	Lys 116 (Nζ)	4.0
	B	*	O 16	Asn 74 (Nδ	4.1		D	**	O 18	Asn 106 (Nδ)	3.3
	C	*	O 9	Lys 1 (Nα)[b]	3.6		D	**	O 21	Asn 106 (Nδ)	3.0
	C	*	O 10	Lys 1 (Nα)[b]	4.1		D	**	O 10	Lys 116 (Nζ)	3.9
	C	*	O 8	Gln 41 (Nε)	3.1		D	**	O 12	Lys 116 (Nζ)	2.5
							D	**	O 19	Lys 116 (Nζ)	3.6
							D	**	O 18	Lys 116 (Nζ)	3.8
							D	**	O 20	Lys 116 (Nζ)	3.6
							D	**	O 21	Lys 116 (Nζ)	3.7
							B	***	O 22	Arg 73 (Nη)	2.7

[a] AU = asymmetric unit, this column indicates protein side chain residues from different asymmetric units. * side chain comes from the same asymmetric unit as TEW molecule. ** side chain comes from a second adjacent asymmetric unit. *** side chain comes from a third adjacent asymmetric unit. [b] (Nα) represents the N-terminal nitrogen.

Mean b-factor and refined occupancies for all existing TEW molecules

During final refinement steps (using PHENIX) TEW molecules were inserted in the electron density followed by refinement of the anomalous signals, occupancies and individual b-factors for several rounds.

Table S2. Mean b-factors and refined occupancies for all TEW molecules.

TEW	Mean b-factor (Å^2)[a]	Refined occupancies[b]
1	34	1.00
2	35	0.68
3	35	0.44
4	53	0.28
5	97	0.76
6	48	0.37
7	204	0.76
8	71	0.32

[a] b-factor describes the displacement of the atomic positions from an average value (information about the flexibility). [b] occupancy gives information about the presence of alternative conformations and their occupation with 1.00 indicating only one conformation, occupancies were refined with phenix.refine[1] using different starting occupancies.

Ball and stick represenation of TEW

Figure S1 is provided for a better understanding of Table S1.

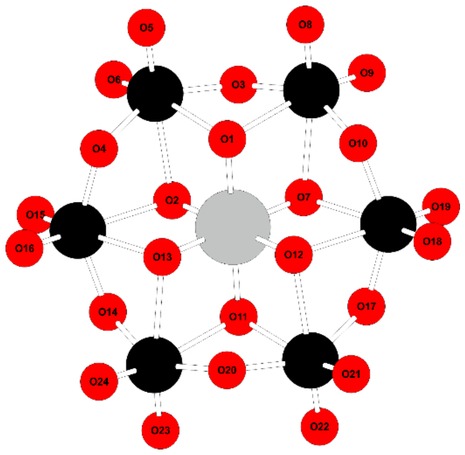

Figure S1. Ball and stick representation of TEW. The oxygen atoms are numbered for a better understanding of the interactions summarized in Table 2 (Colour code: tellurium, grey; tungsten, black; oxygen, red).

Zeta potential measurements

For the zeta potential measurements a Zetasizer Nano ZS (Malvern, United Kingdom) was used. A series of solutions was prepared with a constant HEWL concentration of 4 mg ml^{-1} (280 µM) and increasing TEW concentration in a range from 0 - 1.5 mM. The solutions were buffered in NaOAc (5 mM, pH 4.5). The Zetasizer was adjusted as reported for the zeta potential determination of HEWL with Hofmeister anions.[2] Every solution was measured ten times at a temperature of 25°C and the mean value was used as final data. After the experiment the pH of every solution was measured indicating that TEW did not change the pH significantly (4.97 - 5.05). A possible explanation for the negative zeta potential is the presence of the negative charge on the polyoxometalates.[3][4]

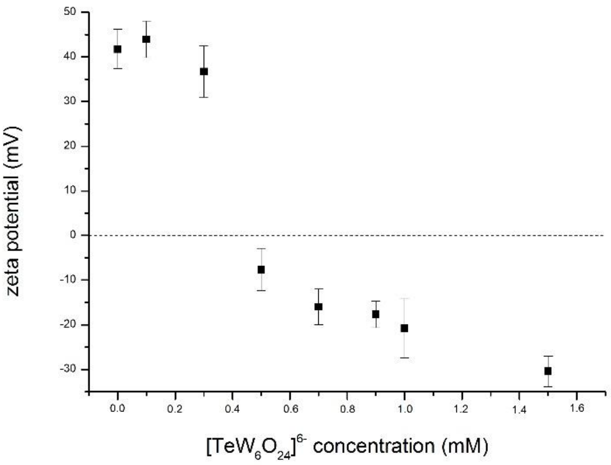

Figure S2. Zeta potential measuremnt. Squares represent dataponts and bars the standard deciation of the measured data.

Reference

[1] P.D. Adams, P.V. Afonine, G. Bunkóczi, V.B. Chen, I.W. Davis, N. Echols, J.J. Headd, L.-W. Hung, G.J. Kapral, R.W. Grosse-Kunstleve, A.J. McCoy, N.W. Moriarty, R. Oeffner, R.J. Read, D.C. Richardson, J.S. Richardson, T.C. Terwilliger, P.H. Zwart, *Acta Crystallogr. D Biol. Crystallogr.* **2010**, *66*, 213-221.

[2] A. Salis, F. Cugia, D.F. Parsons, B.W. Ninham, M. Monduzzi, *Phys. Chem. Chem. Phys. PCCP* **2012**, *14*, 4343-4346.

[3] V. Ball, C. Ringwald, J. Bour, M. Michel, R. Al-Oweini, U. Kortz, *J. Colloid Interface Sci.* **2013**, *409*, 166-173.

[4] H.S. Shah, R. Al-Oweini, A. Haider, U. Kortz, J. Iqbal, *Toxicol. Rep.* **2014**, *1*, 341-352.
